# Insights into early stage of antibiotic development in small- and medium-sized enterprises: a survey of targets, costs, and durations

**DOI:** 10.1186/s40545-018-0135-0

**Published:** 2018-04-05

**Authors:** Christine Årdal, Enrico Baraldi, Ursula Theuretzbacher, Kevin Outterson, Jens Plahte, Francesco Ciabuschi, John-Arne Røttingen

**Affiliations:** 1 Norwegian Institute of Public Health, Postboks 4404 Nydalen, 0403 Oslo, Norway; 2 Uppsala University, Box 513, 751 20 Uppsala, Sweden; 3 Center for Anti-Infective Agents, Vienna, Austria; 4 Boston University, 765 Commonwealth Avenue, Boston, MA 02215 USA; 5 Norwegian Institute of Public Health, Postboks 4404 Nydalen, 0403 Oslo, Norway; 6 Uppsala University, Box 513, 751 20 Uppsala, Sweden; 7 Norwegian Institute of Public Health, University of Oslo, Postboks 4404 Nydalen, 0403, Boks 1072 Blindern, 0316 Oslo, Norway

**Keywords:** Antimicrobial innovation, Antibacterial innovation, DRIVE-AB, Pharmaceutical research and development

## Abstract

**Background:**

Antibiotic innovation has dwindled to dangerously low levels in the past 30 years. Since resistance continues to evolve, this innovation deficit can have perilous consequences on patients. A number of new incentives have been suggested to stimulate greater antibacterial drug innovation. To design effective solutions, a greater understanding is needed of actual antibiotic discovery and development costs and timelines. Small and medium-sized enterprises (SMEs) undertake most discovery and early phase development for antibiotics and other drugs. This paper attempts to gather a better understanding of SMEs’ targets, costs, and durations related to discovery and early phase development of antibacterial therapies.

**Methods:**

DRIVE-AB, a project focused on developing new economic incentives to stimulate antibacterial innovation, held a European stakeholder meeting in February 2015. All SMEs invited to this meeting (*n* = 44) were subsequently sent a survey to gather more data regarding their areas of activity, completed and expected development costs and timelines, and business models.

**Results:**

Twenty-five companies responded to the survey. Respondents were primarily small companies each focusing on developing 1 to 3 new antibiotics, focused on pathogens of public health importance. Most have not yet completed any clinical trials. They have reported ranges of discovery and development out-of-pocket costs that appear to be less expensive than other studies of general pharmaceutical research and development (R&D) costs. The duration ranges reported for completing each phase of R&D are highly variable when compared to previously published general pharmaceutical innovation average durations. However, our sample population is small and may not be fully representative of all relevant antibiotic SMEs.

**Conclusions:**

The data collected by this study provide important insights and estimates about R&D in European SMEs focusing on antibiotics, which can be combined with other data to design incentives to stimulate antibacterial innovation. The variation implies that costs and durations are difficult to generalize due to the unique characteristics of each antibiotic project and depend on individual business strategies and circumstances.

**Electronic supplementary material:**

The online version of this article (10.1186/s40545-018-0135-0) contains supplementary material, which is available to authorized users.

## Background

The world is facing an emerging threat of greater antibiotic resistance [1]. New antibacterial technologies are needed to treat pathogens as they become increasingly resistant to existing antibiotics [1, 2]. Yet, the last new classes of antibiotics to meet unmet needs were discovered in the 1980s [3, 4]. Only about five large pharmaceutical companies invest in antibacterial research & development (R&D) today [5]. However, many more small to medium-sized enterprises (SMEs) have been contributing to the R&D pipeline in this field and are currently the most significant participants in discovery and pre-clinical development activities [6, 7]. Seven out of the eight most recently approved antibiotics were based on key research and early development performed at SMEs [8]. Thus SMEs are key actors in any scheme to reinvigorate antibacterial drug innovation.

Antibacterial innovation is receiving significant political attention of late, including in the G7 and G20 groups of countries [9], the World Health Organization [10] and the United Nations General Assembly [11]. The United Kingdom has provided political momentum to increase antibacterial innovation by commissioning the AMR Review, led by the economist, Lord Jim O’Neill, to propose potential solutions, which were completed in May 2016 [12]. Europe’s Innovative Medicines Initiative (IMI) has financed a project, DRIVE-AB (i.e., Driving reinvestment in research and development for antibiotics and advocating their responsible use, www.drive-ab.eu), a consortium of 16 public sector partners and seven pharmaceutical companies, which aimed to transform the way policymakers stimulate innovation, sustainable use and equitable access of novel antibacterial products to meet public health needs. This article is a part of DRIVE-AB’s research efforts.

A variety of economic incentives have been proposed to stimulate antibacterial drug innovation [12–15]. Since many large pharmaceutical companies have exited the antibiotic field citing unsatisfactory commercial returns [16], the incentives are aimed at stimulating greater private sector involvement by increasing publicly sponsored rewards at the time of regulatory approval so that antibacterial innovation becomes an attractive business case. Determining the appropriate reward amount is a challenging task since it needs to sway innovators and investors to increase their private investments in antibacterial R&D while at the same time ensuring that the public sector is receiving value for money and meeting the important public health goals of sustainable use and equitable access. Knowledge on R&D costs, timelines, and profit expectations of pharmaceutical and venture capital companies is important in order to design and scale up effective solutions. Such knowledge about SMEs is of particular importance, given their position as the primary early-stage antibiotic innovators.

Actual pharmaceutical R&D costs are deemed highly confidential and controversial. Few researchers have been allowed access to this type of data, with the one main exception of DiMasi and colleagues at Tufts University, whose results are based upon data from ten, large pharmaceutical companies focusing on a range of therapeutic areas [17]. The study has been subject to much debate due to a lack of transparency and the resulting implications for pricing of pharmaceuticals [18].

This article is meant to shed some light on the targets, costs and durations of early phase development (up to Phase II clinical trials) for antibiotic innovation (see Table 1) in European SMEs.

**Table 1:** Phases of R&D and Technology Readiness Levels (TRLs) in drug development

R&D Phase	Description	TRL
Research - Discovery activities, hit generation and testing	Generation of chemical starting points (hits) from screens or other drug discovery strategies	2
Research - Lead compound identification	Hits are evaluated and undergo limited optimization to identify promising lead compounds with meaningful activity against the target pathogens and possess the properties needed to make an effective and safe drug	3
Research - Lead compound optimization	Modifying and testing lead compound series to improve compound properties; selecting a candidate drug for further preclinical studies	4
Development - Preclinical testing	Conducting required toxicity and efficacy *in vitro* and *vivo* studies under good laboratory practice (GLP) protocols, and chemistry, manufacturing and control (CMC) studies	5
Development - Phase I clinical trials	Testing the candidate drug in healthy volunteers to determine pharmacokinetics, safe dose ranges and identify common toxicity; pharmacokinetic data feed into pharmacokinetic/ pharmacodynamic (PK/PD) models to determine the most appropriate doses for the next phase	6
Development - Phase II clinical trials	Testing the candidate drug in a small number of patients to obtain preliminary efficacy data and more short term safety information; refining PK/PD models	7
Development - Phase III clinical trials	Testing on a larger number of patients to document efficacy, determine non-inferiority activity (or rarely superiority) and safety compared to other indicated drugs	8

## Methods

Forty-four (44) European-based SMEs were invited to attend a stakeholder meeting in London in February 2015, with a purpose to understand the environment in which SMEs operate, their motivations, and the challenges they face in undertaking antibiotic R&D. The list of companies and contacts was gathered through expert advice and personal contacts of all known European SMEs with at least one antibacterial project in their pipelines. Out of the 44 SMEs invited, representatives of twenty-six (26) companies attended the meeting. The companies varied in size, from virtual companies with no full-time employees to those with dozens of employees. The attendees were divided into four groups with rapporteurs assigned to each group. Each group followed a discussion guide which included challenges faced by antibacterial drug-focused SMEs, financial barriers to investment, the role of SMEs in relation to other R&D organizations, and brainstorming on potential incentives to address SME challenges. Findings from the group work were then discussed in plenary. A final meeting report has been produced which includes the discussion questions, as well as the list of the attending SMEs [19].

A survey (see Additional file 1) was sent on March 5, 2015 to these 44 antibacterial-focused SMEs to gather more specific data regarding their areas of activity, development costs and timelines by R&D phase, and business models. Technology Readiness Levels (TRL) [20] are also given in Table 1 in order to ease comparison of this article’s results with other articles. Reminder e-mails were sent on April 8, 2015 and May 5, 2015. Since the survey asks respondents to share confidential information, such as development costs, it was decided that the answers would be best formatted as multiple choice ranges. The intention was to both receive an acceptable response rate as well as to allow participants to complete the survey in about 30 min or less. The ranges for development costs and timelines were based on existing studies from pharmaceutical R&D [17, 21]. Survey participants were asked to select the range that represented the value that it took for the company to complete the identified R&D phase. Therefore, the results for the clinical trials should not be viewed as a value for completing one clinical trial but rather for finishing all of the clinical trials that the company expects to perform for the identified phase for their main antibacterial project. An “antibacterial project” is the R&D surrounding one specific antibiotic candidate or antibacterial technology.

## Results

Twenty-five (25) SMEs responded to our survey (a response rate of 57%). These can be classified mostly as small companies since only one company has more than 100 employees. 54% of the respondents (*n* = 13) had no revenues in 2014. 68% of the respondents (*n* = 17) focused on 1 to 3 internal antibacterial projects. In addition, 40% of the respondents (*n* = 10) outsourced more than half of their R&D budget to external organizations.

The stakeholder meeting identified three main sources of discovery of companies’ antibacterial projects, either they were discovered: (1) in an academic setting which led to the establishment of a spin-off company, (2) independently by an expert in this field who subsequently formed a new company, or (3) in a large pharmaceutical company and subsequently spun-off as an SME. 80% of the survey respondents (*n* = 20) identified their own research as the source of their lead antibacterial project. In a separate question, 20% (*n* = 4) reported that they are spin-offs from universities or research institutes and another 20% (n = 4) spin-offs from a large, multinational pharmaceutical companies.

### Type of products and clinical targets pursued

Companies at the stakeholder meeting expressed that they entered the antibacterial market because there are significant public health opportunities in new antibacterial products with little competition. Several of these stakeholders also claimed that these opportunities are linked to significant unmet public health needs. The research focus of the survey respondents is largely small molecule development, i.e., traditional antibiotics. 76% (*n* = 19) perform R&D for small molecules, and 44% (*n* = 11) focus on antibodies, adjunctive antibacterial technologies including phage-based therapies and preventive vaccines. (Five companies have both small molecules and adjunctive programs.) Most companies focus solely on human health, but 28% (*n* = 5) also target animal health and/or environmental issues. The overwhelming majority of survey respondents claimed that they are involved in research and development of novel products, with 56% (*n* = 14) pursuing a novel class and 20% (n = 5) a novel mode of action.

Participants at the stakeholder meeting agreed that the increasing resistance problem is the primary market opportunity for antibacterial R&D, and SMEs aim, therefore, to meet therapeutic needs caused by emerging resistance against existing antibiotics. 44% (*n* = 11) of respondents stated that the focus of their main antibacterial project is a narrow-spectrum target and 36% (*n* = 9) a pathogen-specific approach. 80% (*n* = 20) of respondents reported their main antibacterial project targets, including *Acinetobacter baumannii*, *Pseudomonas aeruginosa*, *Escherichia coli*, *Klebsiella pneumoniae*, *Neisseria gonorrhoeae*, *Clostridium difficile*, and/or *Staphylococcus aureus*.

SMEs focused on pharmaceutical innovation typically perform discovery and early phase development work (up to Phase II clinical trials). SMEs at the stakeholder meeting stated that they struggled to find an exit strategy or pathways to commercialize their products due to the paucity of large, pharmaceutical companies actively pursuing R&D on antibacterial products. Many did not see a realistic way to commercialize products by themselves, but were considering taking on this role due to the few commercialization options. When asked to select among multiple exit strategies, 71% (*n* = 17) of respondents expressed hope to be acquired, 63% (*n* = 15) expressed hope to out-license their products, and only 17% (*n* = 4) would consider commercializing their products on their own. This in turn translates into their expectations on the extent of their development work. 36% (*n* = 9) of respondents aimed to complete Phase II clinical trials before out-licensing or selling their main antibacterial projects, whereas 24% (*n* = 6) aimed to complete Phase I clinical trials. And 24% (n = 6) aimed to out-license or sell prior to clinical trials.

### Discovery and development costs and timelines

Respondents to the survey were asked to report discovery and development costs concerning three stages of their main antibacterial project – for each completed phase, the current phase, and the next phase. They were requested not to include opportunity cost or the costs of other candidate products. Therefore, the development cost should represent the out-of-pocket cost in order to complete the particular R&D phase for one antibacterial project. They were also asked about the duration for each phase, both completed and current. We were not able to measure the important component of the quality and scope of the work, for example whether the preclinical testing met bare minimum standards or was more extensive in order to better characterize the project.

**Fig. 1:**
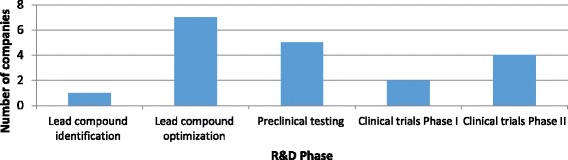
Current phase of R&D for the main antibacterial project

The following development costs and timelines relate only to those who reported performing R&D on small molecules (*n* = 19) in order to report similar activities. Figure 1 shows the current phase of R&D for the main antibacterial project by the SMEs focusing on small molecules.

#### Lead compound identification

Eight SMEs have reported that they have completed lead compound identification. This may seem contradictory since 18 companies have reported that they are currently beyond lead compound identification. We can only presume that these ten companies either chose not to report the data or that they acquired the main antibacterial project after lead compound identification had already been performed. One company, at the time of the survey, was currently performing lead compound identification.

**Fig. 2:**
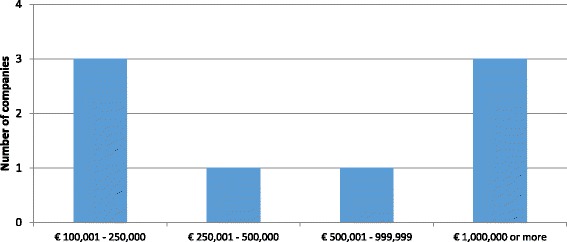
Out-of-pocket cost of lead compound identification per main antibiotic project

All eight companies reported that it took four years or less, including three that reported it took a year or less. Development costs ranged widely from € 100,001–250,000 (*n* = 3) to more than € 1 million (n = 3) (see Fig. 2) with the remaining two companies falling in between these two ranges. The company currently performing lead compound identification at the time of the survey expected it to take in total 6 months to one year and cost less than € 1 million. Three out of the eight claimed that the main antibacterial project represented a novel class and three claimed a novel mode of action. However, neither duration nor the cost of research seemed to have been influenced by novelty, meaning that the novel products were spread across all cost and duration ranges. Therefore, we can estimate that SMEs may spend from € 100,001 to more than € 1,000,000 on lead compound identification which can take as short as 6 months or as long as 4 years.

#### Lead compound optimization

Four companies reported that they have completed lead compound optimization. Seven companies reported, at the time of the survey, that they were currently performing lead compound optimization. One company, at the time of the survey, was performing the previous R&D phase (lead compound identification) and estimated costs for lead compound optimization.

Among the four companies that have completed this phase, one took less than a year, another took one to two years, and the remaining two took two to four years. Amongst the seven companies, at the time of the survey, currently performing this phase, one company expects it to take less than six months, another predicts it will take six months to one year, four expect it to take one to two years, and the remaining company expects two to four years.

**Fig. 3:**
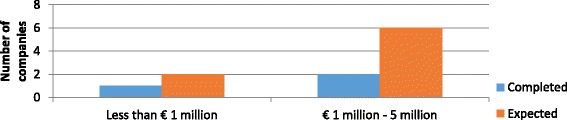
Out-of-pocket costs of lead compound optimization – completed and expected – per main antibiotic project

Three out of the four companies that have completed the phase reported the costs incurred. One reported costs less than € 1 million and the other two companies between € 1–5 million. Among the eight companies with expected costs, six expected that the phase will cost between € 1–5 million, whereas two others estimated less than € 1 million. See Fig. 3 for the completed and expected costs by number of SMEs.

Out of the twelve companies in total reporting for lead compound optimization, seven claimed that the main antibacterial project represented a novel class and four claimed a novel mode of action. (The remaining company was performing R&D on a known class.) However, neither duration nor cost seemed to be influenced by novelty, meaning that the products described as novel were spread across all cost and duration ranges. The majority of SMEs have spent (or plan to spend) from € 1 to 5 million on lead compound optimization which can take as short as 6 months or as long as 4 years.

#### Preclinical testing

Four companies reported that they have completed preclinical testing. Five companies reported, at the time of the survey, that they were currently performing preclinical testing. Seven companies, at the time of the survey, were at the phase of lead compound optimization (before preclinical testing) and therefore estimated the costs for preclinical testing.

Among the four companies that have completed the phase, only three reported time durations. All took from one to two years. Amongst the five companies, at the time of the survey, currently performing the phase, one expects it to take six months to one year, and the remaining four expect it to take one to two years.

**Fig. 4:**
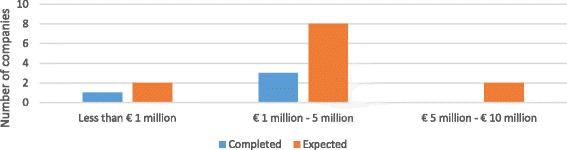
Out-of-pocket costs of preclinical testing – completed and expected – per main antibiotic project

Amongst the four companies that have completed the phase, one reported costs less than € 1 million and the remaining three between € 1–5 million. Among the twelve companies with expected costs (i.e., those currently performing preclinical testing or the previous phase, lead optimization), two expect that the phase will cost less than € 1 million, eight between € 1–5 million, and two between € 5–10 million. See Fig. 4 for the completed and expected costs by number of SMEs.

Out of the 16 companies in total reporting for preclinical testing, nine claimed that the main antibacterial project represented a novel class and three claimed a novel mode of action. (The remaining four companies are performing R&D on known classes.) However, neither duration nor cost seemed to have been influenced by novelty, meaning that the novel products were generally spread across all cost and duration ranges. Both two out of three of the least expensive projects (less than € 1 million) and the two most expensive projects (estimated to cost € 5–10 million) represent novel classes. The shortest duration (6 months to one year) was to complete preclinical testing for a project related to a known class. The majority of SMEs have spent (or plan to spend) from between € 1 and 5 million on preclinical testing which can take approximately one to two years.

#### Phase I clinical trials

Six companies reported that they have completed Phase I clinical trials. Two companies reported, at the time of the survey, that they were currently performing Phase I clinical trials. Five companies, at the time of the survey, were performing the previous R&D phase (preclinical trials) and estimated costs for Phase I clinical trials.

Among the six companies that have completed the phase, three reported that it took six months to a year to complete Phase I clinical trials, two reported between one to two years, with the remaining reporting five or more years. Amongst the two companies, at the time of the survey, currently performing the phase, one expects it to take less than six months and the other six months to a year.

**Fig. 5:**
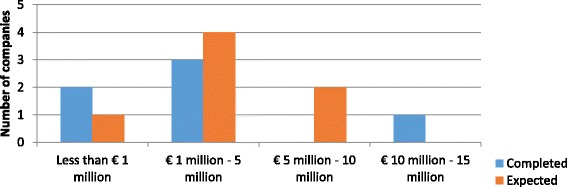
Out-of-pocket costs of the Phase I clinical trial program – completed and expected – per main antibiotic project

Among the six companies that have completed the phase, two reported costs less than € 1 million, three between € 1–5 million, and the remaining between € 10–15 million. Among the seven companies with expected costs, one expects that the phase will cost less than € 1 million, four between € 1–5 million, and two between € 5–10 million. See Fig. 5 for the completed and expected costs by number of SMEs.

Out of the 13 companies in total reporting for Phase I clinical trials, seven claimed that the main antibacterial project represented a novel class and two claimed a novel mode of action. (The remaining four represented R&D on known classes.) However, neither duration nor cost seemed to have been influenced by novelty, meaning that the products described as novel were spread across all cost and duration ranges. The one company that reported a duration of five years or more is developing a novel class. The majority of SMEs have spent (or plan to spend) from € 1 to 10 million on Phase I clinical trials which can take six months to two years.

#### Phase II clinical trials

Only one company has completed Phase II clinical trials. In order to safeguard the anonymity of results since few European antibacterial-related SMEs have completed Phase II clinical trials we have combined the completed and expected figures for Phase II. Three companies, at the time of the survey, were performing Phase II clinical trials, and two companies were performing Phase I clinical trials and reported the estimated costs for Phase II as well.

**Fig. 6:**
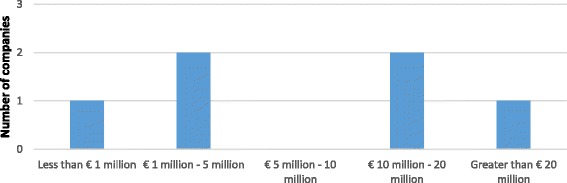
Out-of-pocket costs of Phase II clinical trials – completed and expected – per main antibiotic project

Four companies reported duration data, two reported that it took one to two years to complete Phase II clinical trials and the other two reported between two to four years. Among the six companies reported cost data, one reported costs less than € 1 million, one between € 1–5 million, two between € 10–20 million, and the remaining one more than €20 million (Fig. 6).

Out of the six companies in total reporting for Phase II clinical trials, four claimed that the main antibacterial project represented a novel class and one claimed a novel mode of action. (The remaining company is performing R&D on a known class.) However, neither duration nor cost seemed to have been influenced by novelty, meaning that the products described as novel were generally spread across all cost and duration ranges with the notable exception that the most expensive assertion (more than €20 million) was related to trials for a known class. The majority of SMEs have spent (or plan to spend) from € 1 to 20 million on Phase II clinical trials which can take one to four years.

## Discussion

The results from our stakeholder meeting and survey provided data regarding development targets, development costs and timelines of European antibacterial-focused SMEs. These may not be a full representation of antibacterial SMEs in general since the sample population is small, and it becomes even smaller when the data are further sub-divided by R&D phase. Additionally, our findings report both actual and expected out-of-pocket costs within fairly broad ranges (as indicated in each of the previous figures). Expected costs may change over time. However, the results provided important insights into the therapeutic targets, R&D costs and R&D phase durations of European SMEs operating in antibiotic development.

DRIVE-AB and other initiatives need estimation of cost and duration to be made in order to effectively calculate adequate rewards. An “adequate” reward is one where the publicly-sponsored reward will generate a positive return on investment (or net present value) for the innovator without the public sector over-paying. Based on our results we propose the ranges in Table 2 to estimate development costs of antibiotics for SMEs. (We state antibiotics here since these results focus solely on small molecule R&D.) To generate these ranges, for most values, we have selected the lowest and highest figures reported by the majority of SMEs who claim to be developing novel classes or modes of action. For lead compound identification, the higher boundary value is “more than € 1 million”. This is unfortunate since it does not give us a definitive value as we expected the average value to be smaller when designing the survey. Therefore, our proposed maximum cost is taken from the general pharmaceutical R&D cost fig. [22] which is USD 2.5 million. (The Euro as per the current date of this article is slightly higher than the US dollar. We have given an equivalent in Euros since otherwise this figure appears to give an incorrect impression of precision).

**Table 2:** Proposed minimum and maximum out-of-pocket cost per R&D phase for SME antibiotic innovation for DRIVE-AB models

R&D Phase	General Pharmaceutical R&D Costs	Sertkaya et al. [23]	Proposed Minimum Cost	Proposed Maximum Cost
Lead compound identification	USD 2.5 million [22]	(Used general pharmaceutical cost)	€ 100,000	€ 2.5 million
Lead compound optimization	USD 10 million [22]	(Used general pharmaceutical cost)	€ 1 million	€ 5 million
Preclinical testing	USD 5 million [22]	(Used general pharmaceutical cost)	€ 1 million	€ 5 million
Phase I clinical trials	USD 25 million [17]	USD 10 million	€ 1 million	€ 10 million
Phase II clinical trials	USD 35 million [17]	USD 9–16 million	€ 1 million	€ 20 million

In order to give a perspective regarding our proposed minimum and maximum costs per R&D phase we include two other studies [17, 22] which look across all therapeutic areas (which we call “general pharmaceutical R&D costs”) and the antibacterial-specific costs reported by Sertkaya et al. (Table 2) [23]. Paul et al. utilized industry benchmarking data as well as internal data from the pharmaceutical company, Eli Lilly and Company, therefore, representing large pharmaceutical company costs only [22]. DiMasi et al. utilized data from ten multinational pharmaceutical companies. Sertkaya et al. interviewed about ten experts in antibacterial drug development and company representatives to gather their data regarding costs, in addition to reviewing the published literature [23]. For Phase II clinical trials Sertkaya et al. estimated the cost by indication, therefore, a range is given [23].

Our findings suggest that SMEs expect to perform antibacterial drug development less expensively than large pharmaceutical companies. One interpretation is that SMEs are leaner in structure and more cost-effective in their R&D activities. An alternative explanation may be that SMEs focus mainly on the essential studies that are minimally required by regulatory agencies for entry into limited Phase 2 studies and that they conduct additional studies at later time points. Total costs may be higher than those reported by SMEs here as the company that intends to commercialize the antibiotic may need to back-fill missing data to get licensure. Larger companies may include additional supporting studies or study variables to increase confidence in study results and reduce future risk of failure while a smaller company might be willing to take greater risk while anticipating an exit prior to regulatory approval. Recent changes in the regulatory landscape have significantly influenced costs and duration of later phases of clinical development with the option of abbreviated special pathways with smaller trial sizes. SMEs may also be anticipating these cost savings from regulatory changes; however, these should mostly be related to Phase III clinical trials which are not included in our study. Lastly, as stated clearly throughout the paper, a large percentage of the data is estimated amounts which may vary when faced with real-world obstacles. While the sample companies are inexperienced with performing more advanced development work, at least some SME executives have substantial drug development experience from previous employment.

Table 3 estimates the durations of R&D phases for antibiotic R&D. Again, to generate these ranges for most values we use the higher and lower boundaries of the values reported by the majority of the SMEs claiming to develop either novel classes or modes of action.

**Table 3:** Proposed minimum and maximum durations per R&D phase for SME antibiotic innovation for DRIVE-AB models

R&D Phase	General Pharmaceutical Innovation Durations	Sertkaya et al. [23]	Proposed Minimum Duration	Proposed Maximum Duration
Lead compound identification	1.5 years [22]	(Used general pharmaceutical duration)	6 months	4 years
Lead compound optimization	2 years [22]	(Used general pharmaceutical duration)	6 months	4 years
Preclinical testing	1 year [22]	(Used general pharmaceutical duration)	1 year	2 years
Phase I clinical trials	33 months [17]	0.9 years	6 months	2 years
Phase II clinical trials	39 months [17]	0.8–1.5 years	1 year	4 years

Our duration ranges demonstrate a great deal of variation. The range generally contains the estimates from both the general pharmaceutical innovation and Sertkaya et al., with the exception of Phase I clinical trials. Indeed, in each R&D stage, the duration and costs reported by the surveyed companies vary by a factor of 10 or even more (see e.g., Fig. 5, with costs ranging between €1 million and 10–15 million; or Fig. 6 with a range between 1 and over 20 million). While this large variation implies that that our figures on costs and duration are difficult to generalize, it also suggests that the cost and duration of each antibiotic project depends on many specific factors, such as scientific and technical challenges, the presence or absence of prior data on the specific compound (or compound family), and a range of external factors, including an SME’s availability of funds and experienced leading staff. This variation in durations has also been exemplified in Deak et al., which examined all eight antibiotics approved in the U.S. from 2010 to 2015 [8].

From the stakeholder meeting’s roundtable discussion, several instances emerged of SMEs which were obliged to keep R&D on hold, generally during clinical trials but also in other phases. The barriers reported to cause delays in R&D activities include difficulty in securing funding and partners, preparing initial public offerings, manufacturing and quality control issues.

Regarding targets, both the stakeholder meeting and the survey confirmed that SMEs are focusing their R&D efforts on therapeutic needs caused by emerging resistance against existing antibiotics.

## Conclusions

In conclusion, our results indicate that SMEs seek to deliver antibiotic discovery, preclinical and early clinical development at costs lower than large pharmaceutical companies have reported in the past. Costs for Phase III studies were not assessed. Duration appears to be highly variable but largely in line with other estimates.

DRIVE-AB delivered its final recommendations in January 2018. All recommendations were extensively discussed in consultations with a broad range of stakeholders including policymakers, healthcare insurers (both national and private), medicines regulatory authorities, SMEs, national research funding agencies, academic research institutions, and more. Although principally European in focus, DRIVE-AB actively engaged stakeholders globally to ensure that its recommendations can be integrated in a broader context to ensure sustainable access to efficacious antibiotics and ultimately combatting resistance.

DRIVE-AB performed a computer simulation on different innovation incentives and combined several estimates of costs and durations such as those reviewed in this paper in order to calculate potential profits. It was valuable to have direct data from SMEs in order to develop realistic reward models. Despite the abovementioned limitations, the findings in this paper provide further insights that can help devising more precise policy tools for simulating pharmaceutical innovation taking into account their costs and durations. Similar research regarding SMEs outside of Europe would be beneficial.

## Additional file

Additional file 1: Survey for SMEs. (PDF 447 kb)

## Abbreviations

DRIVE-AB: A research project name - Driving reinvestment in research and development for antibiotics and advocating their responsible use
IMI: The European Union’s Innovative Medicines Initiative
R&D: Research and development
SME: Small and medium-sized enterprises
TRL: Technology Readiness Levels
