# Supplementary material for: Insights into early stage of antibiotic development in small- and medium-sized enterprises: a survey of targets, costs, and durations
Source: J Pharm Policy Pract. 2018 Apr 5;11:8. doi: 10.1186/s40545-018-0135-0 (PMC5885303; doi:10.1186/s40545-018-0135-0)
Supplement: Supplementary file 1 — Survey for SMEs. (PDF 447 kb) [file 40545_2018_135_MOESM1_ESM.pdf]

DRIVE-AB Survey for small and medium biopharmaceutical companies

Thank you for taking approximately 30 minutes to complete this survey.

The data from this survey will be held strictly confidential. Only seven individuals will have access to the raw data. They are: Enrico Baraldi, Francesco Ciabuschi, Carl Anderson Kronlid, Olof Lindahl and Christopher Okhravi (all of Uppsala University) and Jens Plahte and Christine Årdal (of the Norwegian Institute of Public Health). The anonymous, summarized results will be published as well as used as inputs into quantitative simulations to test different incentives. We will send you a copy of the paper when it is published. No organization will be identifiable within the published article.

Your participation, of course, is completely voluntary and has no impact on any interaction with the DRIVE-AB project, its partners, the EU or any government.

In this survey we often refer to your “lead antibacterial project”. Please choose one antibacterial project that has to date been one of your company's primary R&D efforts.

Please start by answering the question below.

1) Can you please indicate the name of the antibacterial company you represent?

[ ]

2) What year was your company established?

[ ]

3) How many full-time equivalent employees does your company employ today?

- ○ 1-5
- ○ 6-10
- ○ 11-50
- ○ 51-100
- ○ More than 100
- ○ We are a virtual company with zero full-time equivalent employees.
- ○ I don't know

4) * What type of antibacterial approach is your company pursuing? Please choose as many cate

- ☐ Small molecule
- ☐ Antibody
- ☐ Preventive vaccine
- ☐ Phage-based
- ☐ Anti-virulence
- ☐ Adjunctive therapy
- ☐ Other [ ]
- ☐ I don't know

5) * Which of the following health applications is your company focusing on?

- ○ Only human health
- ○ Both human and animal health

- Human, animal and environmental health
- Other
- I don't know

6) In 2014 what share of your R&D budget was outsourced to other organizations?

- Less than 25%
- 26-50%
- 51-75%
- More than 75%
- We do not outsource our R&D.
- Other
- I don't know

7) What was the annual revenue of your company in 2014?

- € 1 - 99,000
- € 100,000 - 299,000
- € 300,000 - 1 million
- More than € 1 million
- We do not yet have revenues.
- Other
- I don't know

8) * Is your company a spin-off from a university or research institute?

- Yes
- No
- I don't know

This box is shown in preview only.

The following criteria must be fulfilled for this question to be shown:

- (
  - If "Is your company a spin-off from a university or research institute? " *equals* "Yes"
-)

9) Which university or institute(s)?

This box is shown in preview only.

The following criteria must be fulfilled for this question to be shown:

- (
  - If "Is your company a spin-off from a university or research institute? " *equals* "Yes"
-)

10) Do IP (intellectual property) requirements in relation to this university(ies)/institute(s) rep

- 1 - No obstacles
- 2 - Almost no obstacles
- 3 - Some obstacles
- 4 - Major obstacles
- 5 - Extreme obstacles

○ I don't know

This box is shown in preview only.

The following criteria must be fulfilled for this question to be shown:

- (
    - If "Do IP (intellectual property) requirements in relation to this university (ies)/institute(s) represent an obstacle to pursuing your company goals? " *equals* "2 - Almost no obstacles"
    - or
    - If "Do IP (intellectual property) requirements in relation to this university (ies)/institute(s) represent an obstacle to pursuing your company goals? " *equals* "4 - Major obstacles"
    - or
    - If "Do IP (intellectual property) requirements in relation to this university (ies)/institute(s) represent an obstacle to pursuing your company goals? " *equals* "3 - Some obstacles"
-)

11) In which sense do IP issues represent an obstacle, if applicable?

This box is shown in preview only.

The following criteria must be fulfilled for this question to be shown:

- (
    - If "Is your company a spin-off from a university or research institute? " *equals* "<#na#>I don't know"
    - or
    - If "Is your company a spin-off from a university or research institute? " *equals* "No"
-)

12) Is your company a spin-off from a large, multinational pharmaceutical company?

○ Yes
○ No
○ I don't know

This box is shown in preview only.

The following criteria must be fulfilled for this question to be shown:

- (
    - If "Is your company a spin-off from a large, multinational pharmaceutical company? " *equals* "Yes"
-)

13) Which pharmaceutical company?

This box is shown in preview only.

The following criteria must be fulfilled for this question to be shown:

- (
    - If "Is your company a spin-off from a large, multinational pharmaceutical company? " *equals* "Yes"
-)

14) Do IP (intellectual property) requirements in relation to this pharmaceutical company repres

○ 1 - No obstacles
○ 2 - Almost no obstacles
○ 3 - Some obstacles

○ 4 - Major obstacles
○ 5 - Extreme obstacles
○ I don't know

This box is shown in preview only.

The following criteria must be fulfilled for this question to be shown:

- (
  - If "Do IP (intellectual property) requirements in relation to this pharmaceutical company represent an obstacle to pursuing your company goals? " *equals* "4 - Major obstacles"
  - or
  - If "Do IP (intellectual property) requirements in relation to this pharmaceutical company represent an obstacle to pursuing your company goals? " *equals* "3 - Some obstacles"
  - or
  - If "Do IP (intellectual property) requirements in relation to this pharmaceutical company represent an obstacle to pursuing your company goals? " *equals* "2 - Almost no obstacles"
-)

15) In which sense do IP issues represent an obstacle, if applicable?

16) * How many antibacterial LEADS is your company currently pursuing?

○ 0
○ 1 - 10
○ 11 - 50
○ More than 50
○ Other
○ I don't know

17) * How many antibacterial PROJECTS is your company currently pursuing?

○ 1
○ 2
○ 3
○ More than 3
○ Other
○ I don't know

18) * What is the source of your company's lead antibiotic project? Please check all that apply.

☐ Own research
☐ Academic research
☐ Research from a research institute
☐ Research from another pharmaceutical company
☐ Other
☐ I don't know

19) What share of your company's financial resources in 2014 were dedicated to your lead antib

○ Less than 25%
○ 26 - 50%
○ 51 - 75%

- More than 75%
- Other
- I don't know

20) Is your company's lead antibacterial project taking a broad or a narrow spectrum approach?

- Broad spectrum (both gram positive AND gram negative)
- Narrow spectrum (gram-positive OR gram-negative)
- Pathogen specific
- Too early to know
- Not applicable
- Other
- I don't know

This box is shown in preview only.

The following criteria must be fulfilled for this question to be shown:

- (
  - If "Is your company's lead antibacterial project taking a broad or a narrow spectrum approach? " *equals* "Broad spectrum (both gram positive AND gram negative)"
-)

21) Please specify the range of this spectrum.

This box is shown in preview only.

The following criteria must be fulfilled for this question to be shown:

- (
  - If "Is your company's lead antibacterial project taking a broad or a narrow spectrum approach? " *equals* "Narrow spectrum (gram-positive OR gram-negative)"
  - or
  - If "Is your company's lead antibacterial project taking a broad or a narrow spectrum approach? " *equals* "Pathogen specific"
-)

22) Please specify which pathogen(s) it is targeting.

23) What degree of novelty does your lead antibacterial project represent?

- A novel class
- Novel mode of action
- Known class
- Other
- I don't know

24) * Does the development and commercial evaluation of your lead antibacterial project assum

- ○ Yes
- ○ No
- ○ Ideally
- ○ Other [ ]
- ○ I don't know

25) Does your lead antibacterial project focus on:

- ○ Non-resistant bacteria
- ○ Resistant bacteria that have not yet reached the level of multi-drug resistance
- ○ Multi-drug resistant bacteria
- ○ Other [ ]
- ○ I don't know

26) * Which market segment is your company targeting? Please select all that apply.

- ☐ Community / General Practitioner
- ☐ Hospital
- ☐ Other [ ]
- ☐ I don't know

27) What stage does your company AIM to COMPLETE before out-licensing or selling your lead a

- ○ Lead compound optimization
- ○ Preclinical testing
- ○ Clinical trials Phase I
- ○ Clinical trials Phase II
- ○ Clinical trials Phase III
- ○ Regulatory approval
- ○ We intend to commercialize the product.
- ○ Other [ ]
- ○ I don't know

28) Which stages has your company ALREADY completed for its lead antibacterial project? Pleas

- ☐ Lead compound identification
- ☐ Lead compound optimization
- ☐ Preclinical testing
- ☐ Clinical trials Phase I
- ☐ Clinical trials Phase II
- ☐ Clinical trials Phase III
- ☐ Regulatory approval
- ☐ Other [ ]
- ☐ I don't know

This box is shown in preview only.

The following criteria must be fulfilled for this question to be shown:

- (
  - If "Which stages has your company ALREADY completed for its lead antibacterial project? Please check all that apply." *equals* "Lead compound identification "
-)

29) How long did it take your company to complete Lead Compound Identification for your lead a

- ○ Less than 6 months
- ○ 6 months – 1 year
- ○ 1 year – 2 years
- ○ 2 – 4 years
- ○ 5 years or more
- ○ Other
- ○ I don't know

This box is shown in preview only.

The following criteria must be fulfilled for this question to be shown:

- (
  - If “Which stages has your company ALREADY completed for its lead antibacterial project? Please check all that apply.” *equals* “Lead compound identification ”
-)

30) Compared to your company expectations at the beginning of Lead Compound Identification,

- ○ 1 - Much shorter than expected
- ○ 2 - Slightly shorter than expected
- ○ 3 - As long as expected
- ○ 4 - Slightly longer than expected
- ○ 5 - Much longer than expected
- ○ Other
- ○ I don't know

This box is shown in preview only.

The following criteria must be fulfilled for this question to be shown:

- (
  - If “Which stages has your company ALREADY completed for its lead antibacterial project? Please check all that apply.” *equals* “Lead compound identification ”
-)

31) How much did it cost your company to complete Lead Compound Identification for your lead

- ○ Less than € 100,000
- ○ € 100,001 - € 250,000
- ○ € 250,001 - € 500,000
- ○ € 500,001 - € 999,999
- ○ More than € 1,000,000
- ○ Other
- ○ I don't know

This box is shown in preview only.

The following criteria must be fulfilled for this question to be shown:

- (
  - If “Which stages has your company ALREADY completed for its lead antibacterial project? Please check all that apply.” *equals* “Lead compound identification ”
-)

32) Compared to your company expectations at the beginning of Lead Compound Identification,

- ○ 1 - Much less than expected
- ○ 2 - Slightly less than expected

- 3 - As much as expected
- 4 - Slightly more than expected
- 5 - Much more than expected
- Other
- I don't know

This box is shown in preview only.

The following criteria must be fulfilled for this question to be shown:

- (
  - If "Which stages has your company ALREADY completed for its lead antibacterial project? Please check all that apply." *equals* "Lead compound optimization "
-)

33) How long did it take your company to complete Lead Compound Optimization for your lead a

- Less than 6 months
- 6 months – 1 year
- 1 year – 2 years
- 2 – 4 years
- 5 years or more
- Other
- I don't know

This box is shown in preview only.

The following criteria must be fulfilled for this question to be shown:

- (
  - If "Which stages has your company ALREADY completed for its lead antibacterial project? Please check all that apply." *equals* "Lead compound optimization "
-)

34) Compared to your company expectations at the beginning of Lead Compound Optimization, t

- 1 - Much shorter than expected
- 2 - Slightly shorter than expected
- 3 - As long as expected
- 4 - Slightly longer than expected
- 5 - Much longer than expected
- Other
- I don't know

This box is shown in preview only.

The following criteria must be fulfilled for this question to be shown:

- (
  - If "Which stages has your company ALREADY completed for its lead antibacterial project? Please check all that apply." *equals* "Lead compound optimization "
-)

35) How much did it cost your company to complete Lead Compound Optimization for your lead a

- Less than € 1 million
- € 1 million - € 5 million
- € 5 million - € 10 million
- € 10 million - € 15 million
- More than € 15 million
- Other

○ I don't know

This box is shown in preview only.

The following criteria must be fulfilled for this question to be shown:

- (
  - If "Which stages has your company ALREADY completed for its lead antibacterial project? Please check all that apply." *equals* "Lead compound optimization "
-)

36) Compared to your company expectations at the beginning of Lead Compound Optimization, t

○ 1 - Much less than expected
○ 2 - Slightly less than expected
○ 3 - As much as expected
○ 4 - Slightly more than expected
○ 5 - Much more than expected
○ Other
○ I don't know

This box is shown in preview only.

The following criteria must be fulfilled for this question to be shown:

- (
  - If "Which stages has your company ALREADY completed for its lead antibacterial project? Please check all that apply." *equals* "Preclinical testing "
-)

37) How long did it take your company to complete Preclinical Testing for your lead antibacterial

○ Less than 6 months
○ 6 months – 1 year
○ 1 year – 2 years
○ 2 – 4 years
○ 5 years or more
○ Other
○ I don't know

This box is shown in preview only.

The following criteria must be fulfilled for this question to be shown:

- (
  - If "Which stages has your company ALREADY completed for its lead antibacterial project? Please check all that apply." *equals* "Preclinical testing "
-)

38) Compared to your company expectations at the beginning of Preclinical Testing, the actual d

○ 1 - Much shorter than expected
○ 2 - Slightly shorter than expected
○ 3 - As long as expected
○ 4 - Slightly longer than expected
○ 5 - Much longer than expected
○ Other
○ I don't know

This box is shown in preview only.

The following criteria must be fulfilled for this question to be shown:

- (
  - If "Which stages has your company ALREADY completed for its lead antibacterial project? Please check all that apply." *equals* "Preclinical testing "
-)

39) How much did it cost your company to complete Preclinical Testing for your lead antibacteria

- ○ Less than € 1 million
- ○ € 1 million - € 5 million
- ○ € 5 million - € 10 million
- ○ € 10 million - € 15 million
- ○ More than € 15 million
- ○ Other
- ○ I don't know

This box is shown in preview only.

The following criteria must be fulfilled for this question to be shown:

- (
  - If "Which stages has your company ALREADY completed for its lead antibacterial project? Please check all that apply." *equals* "Preclinical testing "
-)

40) Compared to your company expectations at the beginning of Preclinical Testing, the actual c

- ○ 1 - Much less than expected
- ○ 2 - Slightly less than expected
- ○ 3 - As much as expected
- ○ 4 - Slightly more than expected
- ○ 5 - Much more than expected
- ○ Other
- ○ I don't know

This box is shown in preview only.

The following criteria must be fulfilled for this question to be shown:

- (
  - If "Which stages has your company ALREADY completed for its lead antibacterial project? Please check all that apply." *equals* "Clinical trials Phase I"
-)

41) How long did it take your company to complete Clinical Trials Phase I for your lead antibacte

- ○ Less than 6 months
- ○ 6 months – 1 year
- ○ 1 year – 2 years
- ○ 2 – 4 years
- ○ 5 years or more
- ○ Other
- ○ I don't know

This box is shown in preview only.

The following criteria must be fulfilled for this question to be shown:

- (
  - If "Which stages has your company ALREADY completed for its lead antibacterial project? Please check all that apply." *equals* "Clinical trials Phase I"
-)

42) Compared to your company expectations at the beginning of Clinical Trials Phase I, the actua

- 1 - Much shorter than expected
- 2 - Slightly shorter than expected
- 3 - As long as expected
- 4 - Slightly longer than expected
- 5 - Much longer than expected
- Other
- I don't know

This box is shown in preview only.

The following criteria must be fulfilled for this question to be shown:

- (
  - If "Which stages has your company ALREADY completed for its lead antibacterial project? Please check all that apply." *equals* "Clinical trials Phase I"
-)

43) How much did it cost your company to complete Clinical Trials Phase I for your lead antibact

- Less than € 1 million
- € 1 million - € 5 million
- € 5 million - € 10 million
- € 10 million - € 15 million
- More than € 15 million
- Other
- I don't know

This box is shown in preview only.

The following criteria must be fulfilled for this question to be shown:

- (
  - If "Which stages has your company ALREADY completed for its lead antibacterial project? Please check all that apply." *equals* "Clinical trials Phase I"
-)

44) Compared to your company expectations at the beginning of Clinical Trials Phase I, the actu

- 1 - Much less than expected
- 2 - Slightly less than expected
- 3 - As much as expected
- 4 - Slightly more than expected
- 5 - Much more than expected
- Other
- I don't know

This box is shown in preview only.

The following criteria must be fulfilled for this question to be shown:

- (
  - If "Which stages has your company ALREADY completed for its lead antibacterial project? Please check all that apply." *equals* "Clinical trials Phase II"
-)

45) How long did it take your company to complete Clinical Trials Phase II for your lead antibact

- Less than 6 months
- 6 months – 1 year
- 1 year – 2 years
- 2 – 4 years

○ 5 years or more

○ Other

○ I don't know

This box is shown in preview only.

The following criteria must be fulfilled for this question to be shown:

- (
  - If "Which stages has your company ALREADY completed for its lead antibacterial project? Please check all that apply." *equals* "Clinical trials Phase II"
-)

46) Compared to your company expectations at the beginning of Clinical Trials Phase II, the actu

○ 1 - Much shorter than expected

○ 2 - Slightly shorter than expected

○ 3 - As long as expected

○ 4 - Slightly longer than expected

○ 5 - Much longer than expected

○ Other

○ I don't know

This box is shown in preview only.

The following criteria must be fulfilled for this question to be shown:

- (
  - If "Which stages has your company ALREADY completed for its lead antibacterial project? Please check all that apply." *equals* "Clinical trials Phase II"
-)

47) How much did it cost your company to complete Clinical Trials Phase II for your lead antibac

○ Less than € 1 million

○ € 1 million - € 5 million

○ € 5 million - € 10 million

○ € 10 million - € 20 million

○ More than € 20 million

○ Other

○ I don't know

This box is shown in preview only.

The following criteria must be fulfilled for this question to be shown:

- (
  - If "Which stages has your company ALREADY completed for its lead antibacterial project? Please check all that apply." *equals* "Clinical trials Phase II"
-)

48) Compared to your company expectations at the beginning of Clinical Trials Phase II, the actu

○ 1 - Much less than expected

○ 2 - Slightly less than expected

○ 3 - As much as expected

○ 4 - Slightly more than expected

○ 5 - Much more than expected

○ Other

○ I don't know

This box is shown in preview only.

The following criteria must be fulfilled for this question to be shown:

- (
  - If “Which stages has your company ALREADY completed for its lead antibacterial project? Please check all that apply.” *equals* “Clinical trials Phase III”
-)

49) How long did it take your company to complete Clinical Trials Phase III for your lead antibac

- ○ Less than 6 months
- ○ 6 months – 1 year
- ○ 1 year – 2 years
- ○ 2 – 4 years
- ○ 5 years or more
- ○ Other
- ○ I don't know

This box is shown in preview only.

The following criteria must be fulfilled for this question to be shown:

- (
  - If “Which stages has your company ALREADY completed for its lead antibacterial project? Please check all that apply.” *equals* “Clinical trials Phase III”
-)

50) Compared to your company expectations at the beginning of Clinical Trials Phase III, the act

- ○ 1 - Much shorter than expected
- ○ 2 - Slightly shorter than expected
- ○ 3 - As long as expected
- ○ 4 - Slightly longer than expected
- ○ 5 - Much longer than expected
- ○ Other
- ○ I don't know

This box is shown in preview only.

The following criteria must be fulfilled for this question to be shown:

- (
  - If “Which stages has your company ALREADY completed for its lead antibacterial project? Please check all that apply.” *equals* “Clinical trials Phase III”
-)

51) How much did it cost your company to complete Clinical Trials Phase III for your lead antiba

- ○ Less than € 10 million
- ○ € 10 million - € 25 million
- ○ € 25 million - € 50 million
- ○ € 50 million - € 100 million
- ○ More than € 100 million
- ○ Other
- ○ I don't know

This box is shown in preview only.

The following criteria must be fulfilled for this question to be shown:

- (

- If "Which stages has your company ALREADY completed for its lead antibacterial project? Please check all that apply." *equals* "Clinical trials Phase III"
-)

52) Compared to your company expectations at the beginning of Clinical Trials Phase III, the act

- 1 - Much less than expected
- 2 - Slightly less than expected
- 3 - As much as expected
- 4 - Slightly more than expected
- 5 - Much more than expected
- Other
- I don't know

This box is shown in preview only.

The following criteria must be fulfilled for this question to be shown:

- (
  - If "Which stages has your company ALREADY completed for its lead antibacterial project? Please check all that apply." *equals* "Regulatory approval"
-)

53) How long did it take your company to complete Regulatory Approval for your lead antibacter

- Less than 6 months
- 6 months – 1 year
- 1 year – 2 years
- 2 – 4 years
- 5 years or more
- Other
- I don't know

This box is shown in preview only.

The following criteria must be fulfilled for this question to be shown:

- (
  - If "Which stages has your company ALREADY completed for its lead antibacterial project? Please check all that apply." *equals* "Regulatory approval"
-)

54) Compared to your company expectations at the beginning of Regulatory Approval, the actual

- 1 - Much shorter than expected
- 2 - Slightly shorter than expected
- 3 - As long as expected
- 4 - Slightly longer than expected
- 5 - Much longer than expected
- Other
- I don't know

This box is shown in preview only.

The following criteria must be fulfilled for this question to be shown:

- (
  - If "Which stages has your company ALREADY completed for its lead antibacterial project? Please check all that apply." *equals* "Regulatory approval"
-)

55) How much did it cost your company to complete Regulatory Approval for your lead antibacte

- ○ Less than € 1 million
- ○ € 1 million - € 2 million
- ○ € 2 million - € 3 million
- ○ € 3 million - € 4 million
- ○ More than € 4 million
- ○ Other
- ○ I don't know

This box is shown in preview only.

The following criteria must be fulfilled for this question to be shown:

- (
  - If "Which stages has your company ALREADY completed for its lead antibacterial project? Please check all that apply." *equals* "Regulatory approval"
-)

56) Compared to your company expectations at the beginning of Regulatory Approval, the actual

- ○ 1 - Much less than expected
- ○ 2 - Slightly less than expected
- ○ 3 - As much as expected
- ○ 4 - Slightly more than expected
- ○ 5 - Much more than expected
- ○ Other
- ○ I don't know

57) * What stage is your company currently in for your lead antibacterial project?

- ○ Lead compound identification
- ○ Lead compound optimization
- ○ Preclinical testing
- ○ Clinical trials Phase I
- ○ Clinical trials Phase II
- ○ Clinical trials Phase III
- ○ Regulatory approval
- ○ Other
- ○ I don't know

This box is shown in preview only.

The following criteria must be fulfilled for this question to be shown:

- (
  - If "What stage is your company currently in for your lead antibacterial project?" *equals* "Lead compound identification "
  - or
  - If "What stage is your company currently in for your lead antibacterial project?" *equals* "Preclinical testing "
  - or
  - If "What stage is your company currently in for your lead antibacterial project?" *equals* "Lead compound optimization "
  - or
  - If "What stage is your company currently in for your lead antibacterial project?" *equals* "Regulatory approval"
  - or
  - If "What stage is your company currently in for your lead antibacterial project?" *equals* "Clinical trials Phase II"
  - or
  - If "What stage is your company currently in for your lead antibacterial project?" *equals* "Clinical trials Phase III"
  - or
  - If "What stage is your company currently in for your lead antibacterial project?" *equals* "Clinical trials Phase I"
-)

58) How long do you expect this stage will take to complete from start to finish?

- ○ Less than 6 months
- ○ 6 months – 1 year
- ○ 1 year – 2 years
- ○ 2 – 4 years
- ○ More than 5 years
- ○ Other [ ]
- ○ I don't know

This box is shown in preview only.

The following criteria must be fulfilled for this question to be shown:

- (
  - If "What stage is your company currently in for your lead antibacterial project?" *equals* "Lead compound optimization "
  - or
  - If "What stage is your company currently in for your lead antibacterial project?" *equals* "Lead compound identification "
  - or
  - If "What stage is your company currently in for your lead antibacterial project?" *equals* "Preclinical testing "
  - or
  - If "What stage is your company currently in for your lead antibacterial project?" *equals* "Regulatory approval"
  - or
  - If "What stage is your company currently in for your lead antibacterial project?" *equals* "Clinical trials Phase II"
  - or
  - If "What stage is your company currently in for your lead antibacterial project?" *equals* "Clinical trials Phase III"
  - or
  - If "What stage is your company currently in for your lead antibacterial project?" *equals* "Clinical trials Phase I"
-)

59) How much do you expect this stage will cost to complete from start to finish?

- ○ Less than € 1 million
- ○ € 1 million - € 5 million
- ○ € 5 million - € 10 million
- ○ € 10 million - € 20 million
- ○ More than € 20 million
- ○ Other [ ]
- ○ I don't know

This box is shown in preview only.

The following criteria must be fulfilled for this question to be shown:

- (
  - If "What stage is your company currently in for your lead antibacterial project?" *equals* "Lead compound identification "
  - or
  - If "What stage is your company currently in for your lead antibacterial project?" *equals* "Lead compound optimization "
  - or
  - If "What stage is your company currently in for your lead antibacterial project?" *equals* "Clinical trials Phase III"
  - or
  - If "What stage is your company currently in for your lead antibacterial project?" *equals* "Clinical trials Phase I"
  - or
  - If "What stage is your company currently in for your lead antibacterial project?" *equals* "Clinical trials Phase II"
  - or
  - If "What stage is your company currently in for your lead antibacterial project?" *equals* "Preclinical testing "
-)

60) What do you believe is the success rate that your company will transition to the next phase?

- ○ 0 - 25 %
- ○ 26 - 50 %

- 51 - 75 %
- More than 75 %
- Other
- I don't know

This box is shown in preview only.

The following criteria must be fulfilled for this question to be shown:

- (
  - If "What do you believe is the success rate that your company will transition to the next phase? " *equals* "26 - 50 %"
  - or
  - If "What do you believe is the success rate that your company will transition to the next phase? " *equals* "0 - 25 %"
  - or
  - If "What do you believe is the success rate that your company will transition to the next phase? " *equals* "More than 75 %"
  - or
  - If "What do you believe is the success rate that your company will transition to the next phase? " *equals* "51 - 75 %"
-)

61) What is the basis for this success rate? Please check all that apply.

- Previous experience
- Industry standard for our current phase
- Company standard for our current phase
- Analogs from other companies
- Other
- I don't know

62) How much do you expect the NEXT stage of development will cost to complete from start to

- Less than € 1 million
- € 1 million - € 5 million
- € 5 million - € 10 million
- € 10 million - € 20 million
- More than € 20 million
- Other
- I don't know

63) How much total external financing has your company received to date?

- Less than € 500,000
- € 500,000 - € 1 million
- € 1 million - € 5 million
- € 5 million - € 20 million
- € 20 million - € 50 million
- More than € 50 million
- Other
- I don't know

This box is shown in preview only.

The following criteria must be fulfilled for this question to be shown:

- (
  - If "How much total external financing has your company received to date? " *equals* "Less than € 500,000"
  - or
  - If "How much total external financing has your company received to date? " *equals* "€ 500,000 - € 1 million"
  - or

- If “How much total external financing has your company received to date? ” *equals* “More than € 50 million”
- or
- If “How much total external financing has your company received to date? ” *equals* “€ 5 million - € 20 million”
- or
- If “How much total external financing has your company received to date? ” *equals* “€ 20 million - € 50 million”
- or
- If “How much total external financing has your company received to date? ” *equals* “€ 1 million - € 5 million”

-)

64) Can you please specify how much your company has received in the form of GRANTS?

- ○ Less than € 500,000
- ○ € 500,000 - € 1 million
- ○ € 1 million - € 5 million
- ○ € 5 million - € 20 million
- ○ € 20 million - € 50 million
- ○ More than € 50 million
- ○ Other
- ○ I don't know

This box is shown in preview only.

The following criteria must be fulfilled for this question to be shown:

- (
  - If “How much total external financing has your company received to date? ” *equals* “Less than € 500,000”
  - or
  - If “How much total external financing has your company received to date? ” *equals* “€ 500,000 - € 1 million”
  - or
  - If “How much total external financing has your company received to date? ” *equals* “More than € 50 million”
  - or
  - If “How much total external financing has your company received to date? ” *equals* “€ 5 million - € 20 million”
  - or
  - If “How much total external financing has your company received to date? ” *equals* “€ 20 million - € 50 million”
  - or
  - If “How much total external financing has your company received to date? ” *equals* “€ 1 million - € 5 million”
-)

65) Can you please specify how much your company has received in the form of LOANS?

- ○ Less than € 500,000
- ○ € 500,000 - € 1 million
- ○ € 1 million - € 5 million
- ○ € 5 million - € 20 million
- ○ € 20 million - € 50 million
- ○ More than € 50 million
- ○ Other
- ○ I don't know

This box is shown in preview only.

The following criteria must be fulfilled for this question to be shown:

- (
  - If “How much total external financing has your company received to date? ” *equals* “Less than € 500,000”
  - or
  - If “How much total external financing has your company received to date? ” *equals* “€ 500,000 - € 1 million”
  - or
  - If “How much total external financing has your company received to date? ” *equals* “More than € 50 million”
  - or
  - If “How much total external financing has your company received to date? ” *equals* “€ 5 million - € 20 million”
  - or

- If "How much total external financing has your company received to date? " *equals* "€ 20 million - € 50 million"
- or
- If "How much total external financing has your company received to date? " *equals* "€ 1 million - € 5 million"

-)

66) Can you please specify how much your company has received in the form of EQUITY-DILUTI

- Less than € 500,000
- € 500,000 - € 1 million
- € 1 million - € 5 million
- € 5 million - € 20 million
- € 20 million - € 50 million
- More than € 50 million
- Other
- I don't know

67) What share of equity is still retained today by the company's founders?

- 0 - 25 %
- 26 - 50 %
- 51 - 75 %
- 76 - 99 %
- All equity is still retained by the company's founders.
- Other
- I don't know

68) How long will your current financing support the activities of the company?

- 1 to 6 months
- 7 months to 1 year
- 1 to 3 years
- More than 3 years
- Other
- I don't know

69) Does your company have a specific Net Present Value (NPV) target?

- Yes
- No
- Other
- I don't know

This box is shown in preview only.

The following criteria must be fulfilled for this question to be shown:

- (
  - If "Does your company have a specific Net Present Value (NPV) target? " *equals* "Yes"
-)

70) What is your company's target for NPV?

This box is shown in preview only.

The following criteria must be fulfilled for this question to be shown:

- (
  - If "Does your company have a specific Net Present Value (NPV) target? " *equals* "No"
-)

71) What are your company's reasons for not using a NPV target?

72) How important have the following performance metrics been in securing financing for your c

	1 - Totally unimportant	2 - Somewhat unimportant	3 - Neutral	4 - Somewhat important	5 - Extremely important	I don't know
Profitability (NPV, ROI or other similar indicators)	○	○	○	○	○	○
Time to exit (either IPO or acquisition)	○	○	○	○	○	○
Time to regulatory approval	○	○	○	○	○	○

73) Which exit strategies are your company's owners most strongly pursuing? Please check all tl

- ☐ Exit by IPO
- ☐ Exit by acquisition
- ☐ Exit by out-licensing
- ☐ No exit strategy - We plan to commercialize our products.
- ☐ Other
- ☐ I don't know

74) How important are collaborations with the following partners for your company?

	1 - Totally unimportant	2 - Somewhat unimportant	3 - Neutral	4 - Somewhat important	5 - Extremely important	I don't know
Academic labs or research institutes	○	○	○	○	○	○
Incubators	○	○	○	○	○	○
Contract Research Organizations (CROs)	○	○	○	○	○	○
Large, multinational pharmaceutical companies	○	○	○	○	○	○
Diagnostic companies	○	○	○	○	○	○
Hospitals and clinical centers	○	○	○	○	○	○

75) Has your company ever collaborated with any of the following partners? Please check all tha

- ☐ Academic labs or research institutes
- ☐ Incubators
- ☐ Contract Research Organizations (CROs)
- ☐ Large, multinational pharmaceutical companies
- ☐ Diagnostic companies
- ☐ Hospitals and clinical centers
- ☐ Other
- ☐ I don't know

76) Which of the following incentives would most assist your company in developing new antiba

- ☐ A stronger pipeline of antibiotic candidates coming from academia/research institutes
- ☐ Significant new grant funding from governments, foundations or patient advocacy groups
- ☐ Value-based pricing for antibiotics and antibiotic alternatives
- ☐ Direct grants for pre-clinical and clinical development from Private-Public Partnerships (PPPs), such as BARDA
- ☐ Tax credits for 50% of clinical development costs (fully refundable)
- ☐ Advance market commitments (AMCs) that guarantee a market for a specified product
- ☐ Faster approvals by EMA/FDA
- ☐ Dramatically lower costs of clinical development
- ☐ Significant financial prizes upon registration or for specific milestones
- ☐ Significantly improved reimbursement for new, powerful antibiotics
- ☐ Post-approval payments to recoup the costs of global registration and establishing a manufacturing base
- ☐ 5 years of additional exclusivity after patent expiration (i.e., years 21-25)
- ☐ Patent tolling (extending the patent life to compensate for periods held off the market until resistance to other products develops)
- ☐ Other
- ☐ I don't know

77) Is there anything else that you would like to tell us?

© Copyright www.questback.com. All Rights Reserved.
